# Supplementary figures and images for: Discovery and functional assessment of a novel adipocyte population driven by intracellular Wnt/β-catenin signaling in mammals
Source: eLife. 2022 May 3;11:e77740. doi: 10.7554/eLife.77740 (PMC9064292; doi:10.7554/eLife.77740)

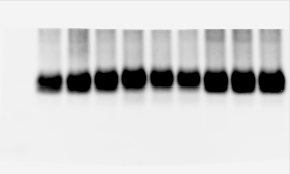

Supplement: Figure 4—source data 1. — Full-scans of western blots. Full-sized image of western blot from Figure 4A and B. Red box indicates area that was cropped and displayed in the indicated figure. [file elife-77740-fig4-data1.zip › Fig.4-source data/Fig.4B/Fig.4B-4E-BP1.tif]

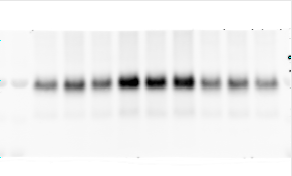

Supplement: Figure 4—source data 1. — Full-scans of western blots. Full-sized image of western blot from Figure 4A and B. Red box indicates area that was cropped and displayed in the indicated figure. [file elife-77740-fig4-data1.zip › Fig.4-source data/Fig.4B/Fig.4B-p-4E-BP1.tif]

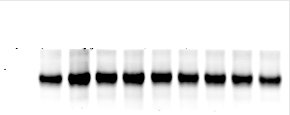

Supplement: Figure 4—source data 1. — Full-scans of western blots. Full-sized image of western blot from Figure 4A and B. Red box indicates area that was cropped and displayed in the indicated figure. [file elife-77740-fig4-data1.zip › Fig.4-source data/Fig.4B/Fig.4B-GSK-3b.tif]

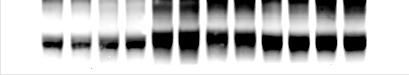

Supplement: Figure 5—source data 1. — Full-scans of western blots. Full-sized image of western blot from Figure 5H. Red box indicates area that was cropped and displayed in the indicated figure. [file elife-77740-fig5-data1.zip › Fig.5-source data/Fig.5H/Fig.5H-b-actin.tif]

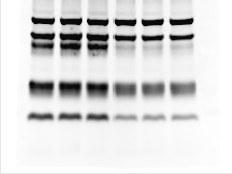

Supplement: Figure 5—figure supplement 3—source data 1. — Full-scans of western blots. Full-sized image of western blot from Figure 5—figure supplement 3B. Red box indicates area that was cropped and displayed in the indicated figure. [file elife-77740-fig5-figsupp3-data1.zip › Fig.5-fig sup3-source data/Figure 5-figure sup 3/OXPHOS-Akita.tif]

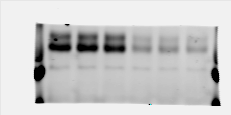

Supplement: Figure 5—figure supplement 3—source data 1. — Full-scans of western blots. Full-sized image of western blot from Figure 5—figure supplement 3B. Red box indicates area that was cropped and displayed in the indicated figure. [file elife-77740-fig5-figsupp3-data1.zip › Fig.5-fig sup3-source data/Figure 5-figure sup 3/UCP1-Akita.tif]

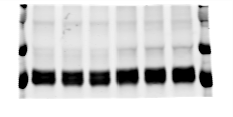

Supplement: Figure 5—figure supplement 3—source data 1. — Full-scans of western blots. Full-sized image of western blot from Figure 5—figure supplement 3B. Red box indicates area that was cropped and displayed in the indicated figure. [file elife-77740-fig5-figsupp3-data1.zip › Fig.5-fig sup3-source data/Figure 5-figure sup 3/actin-Akita.tif]
