# Supplementary material for: Discovery and functional assessment of a novel adipocyte population driven by intracellular Wnt/β-catenin signaling in mammals
Source: eLife. 2022 May 3;11:e77740. doi: 10.7554/eLife.77740 (PMC9064292; doi:10.7554/eLife.77740)
Supplement: Figure 5—source data 1. — Full-scans of western blots. Full-sized image of western blot from Figure 5H. Red box indicates area that was cropped and displayed in the indicated figure. [file elife-77740-fig5-data1.zip › Fig.5-source data/Fig.5-source data1.pdf]

Full-sized Western blot images for Figure 5H

β-actin

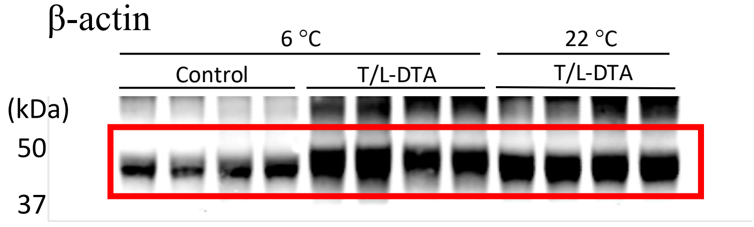

OXPHOS

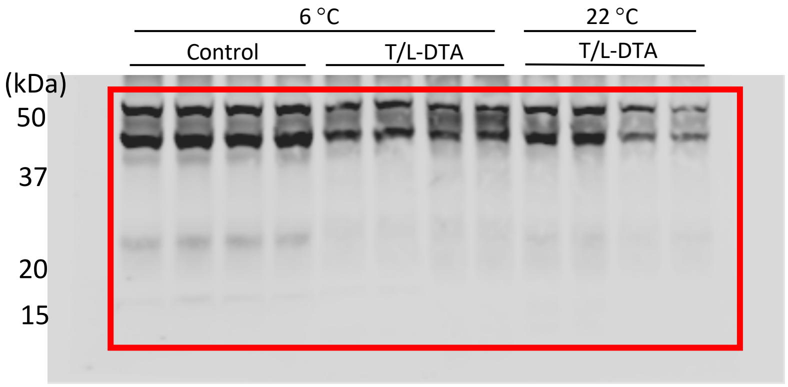

UCP1

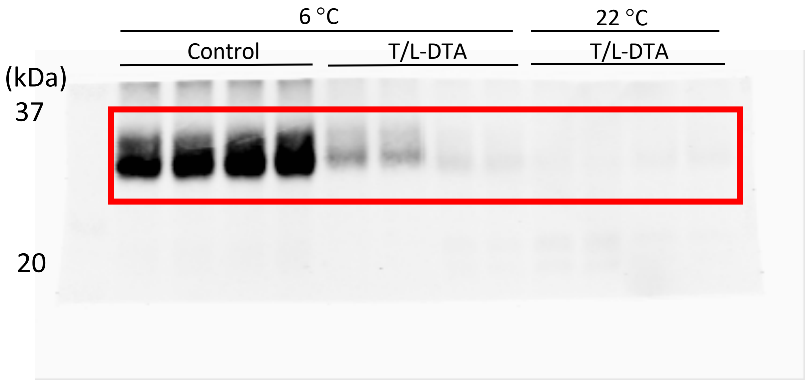

Figure 5-source data 1. Full-scans of Western blots. Full-sized image of Western blot from Figure 5H. Red box indicates area that was cropped and displayed in the indicated figure.
