# Supplementary material for: Discovery and functional assessment of a novel adipocyte population driven by intracellular Wnt/β-catenin signaling in mammals
Source: eLife. 2022 May 3;11:e77740. doi: 10.7554/eLife.77740 (PMC9064292; doi:10.7554/eLife.77740)
Supplement: Supplementary file 1. [file elife-77740-supp1.docx]

**Supplementary table 1.** Primer sequences used for qRT-PCR.

| Gene | Forward | Reverse |
| --- | --- | --- |
| *Ucp1* | AGGCTTCCAGTACCATTAGGT | CTGAGTGAGGCAAAGCTGATTT |
| *Cidea* | TGACATTCATGGGATTGCAGAC | GGCCAGTTGTGATGACTAAGAC |
| *Pgc1a* | AAACTTGCTAGCGGTCCTCA | TGGCTGGTGCCAGTAAGAG |
| *Cox8b* | GAACCATGAAGCCAACGACT | GCGAAGTTCACAGTGGTTCC |
| *Elovl3* | TTCTCACGCGGGTTAAAAATGG | GAGCAACAGATAGACGACCAC |
| *Prdm16* | GGCGAGGAAGCTAGCCAAA | GGTCTCCTCCTCGGCACTCT |
| *Pparg2* | TCGCTGATGCACTGCCTATG | GAGAGGTCCACAGAGCTGATT |
| *Fabp4* | TTCGATGAAATCACCGCAGA | GGTCGACTTTCCATCCCACTT |
| *Adipoq* | TGTTCCTCTTAATCCTGCCCA | CCAACCTGCACAAGTTCCCTT |
| *Cebpa* | CAAGAACAGCAACGAGTACCG | GTCACTGGTCAACTCCAGCAC |
| *Adrb1* | CCGAAAGCAGGTGAATGCAA | AGCCAGTAAGCCATACTAAGCCACA |
| *Adrb2* | CATTGATGTGTTGTGCGTCA | ACTCGGGCCTTATTCTTGGT |
| *Adrb3* | CCTTCCGTCGTCTTCTGTGT | AGCCATCAAACCTGTTGAGC |
| *36B4* | TCCAGGCTTTGGGCATCA | CTTTATCAGCTGCACATCACTCAGA |
